# Plants and Surgery: The Protective Effects of Thymoquinone on Hepatic Injury—A Systematic Review of In Vivo Studies

**DOI:** 10.3390/ijms19041085

**Published:** 2018-04-05

**Authors:** Aysun Tekbas, Jutta Huebner, Utz Settmacher, Uta Dahmen

**Affiliations:** 1 Department of General, Visceral and Vascular Surgery, University of Jena, 07743 Jena, Germany; Utz.Settmacher@med.uni-jena.de (U.S.); Uta.Dahmen@med.uni-jena.de (U.D.); 2 Clinic for Internal Medicine II, Department of Hematology and Internal Oncology, University of Jena, 07743 Jena, Germany; Jutta.Huebner@med.uni-jena.de

**Keywords:** *Nigella sativa*, Thymoquinone, natural remedy, liver, ischemia/reperfusion injury, hepatotoxicity, chemotherapy, hepatic injury

## Abstract

Multimodal treatment concepts including liver transplantation for hepatocellular carcinoma (HCC), extended resection methods and neoadjuvant chemotherapy for colorectal liver metastasis significantly improve patients’ outcome. However, surgery-induced hepatic ischemia-reperfusion injury (IRI) and chemotherapy-associated hepatotoxicity result in hepatocellular damage and compromised liver function. Activation of common key pathways in ischemic liver and hepatotoxic injury results in oxidative stress, inflammatory responses and apoptosis causing organ damage. Controlling liver damage before and during surgery is essential for the postoperative outcome. *Nigella sativa* has a long tradition as a natural remedy. In the essential oil, Thymoquinone (TQ) was identified as the main component and responsible for most of the therapeutic effects. Therefore, this systematic review aimed to summarize the hepatoprotective effects of TQ and its potential suitability to improve surgical outcome by reducing surgical ischemic injury and hepatotoxicity of neoadjuvant chemotherapy. The key findings can be summarized as TQ having strong antioxidant, anti-inflammatory, antifibrotic, anti-/proapoptotic and anticarcinogenic effects. Almost no side effects were reported irrespective of a large dose range, suggesting a wide therapeutic window. These results give rise to the expectation that TQ could evolve to a novel powerful drug to reduce hepatic injury.

## 1. Introduction

### 1.1. Current Treatment Concepts and Hepatotoxicity

Surgical resection is the gold standard for curative treatment of colorectal liver metastasis [1] and hepatocellular carcinoma (HCC) [2]. However, only 10–20% of patients with liver metastasis and <30% with HCC are eligible for surgery due to the tumor size, location and/or the degree of liver function [3,4]. Those patients remarkably benefit from multimodal treatment concepts consisting of neoadjuvant chemotherapy for colorectal liver metastasis [5] as well as from extended resection methods [6] and liver transplantation for HCC [7,8].

Neoadjuvant chemotherapy is applied to downsize the tumor burden in the liver. Reduction of tumor mass may turn a primarily unresectable tumor into a resectable one, enabling a potentially curative resection for an increasing number of patients. Curative resection is the prerequisite for prolonging recurrence-free survival time [9].

Per contra, modern chemotherapy regimens such as Oxaliplatin and Irinotecan are associated with severe damage of the liver parenchyma, mainly leading to sinusoidal obstruction syndrome (SOS) and steatohepatitis [10]. Respectively, both injuries may increase the risk for post-hepatectomy morbidity [11].

The injury in SOS is caused by the damage to endothelial cells in the liver leading to their necrosis and extrusion into sinusoids which results in obstruction and congestion. Furthermore, stellate cells are activated and produce extracellular matrix and collagens. Due to loss of endothelial function, congestion and possibly direct toxicity of the implicated agents, hepatocellular necrosis is caused [10,12,13].

Steatohepatitis is due to an imbalance between triglyceride accumulation and elimination in the liver together with the activation of inflammatory cytokines. This results in interlobular inflammation and ballooning degeneration of hepatocytes [10,14].

#### Transplantation and IRI

In cases of HCC, liver resection can only be offered to patients with little impairment of hepatic function or to those needing only a minor resection. Liver transplantation is a curative option treating the tumor and the underlying liver disease [8]. Modern transplantation medicine includes well-developed surgical techniques, preservation strategies and immunosuppressive drugs making cadaveric or living-donor transplantations possible. Nevertheless, the inevitable hepatic IRI still remains a major problem in liver transplantation, potentially leading to hepatocellular dysfunction, aggravating liver rejection and ultimately contributing to the development of liver failure [15].

The mechanisms involved in the development of IRI are not fully understood. It is a dynamic process consisting of oxidative stress mediated by reactive oxygen species (ROS) leading to pro-inflammatory responses and hepatic damage upon reperfusion [16]. Scavenging of ROS and radicals in ischemic tissues and simultaneous suppression of the activated inflammatory cascade may reduce IRI.

### 1.2. Natural Remedies and Liver Protection

Toxic effects of modern allopathic drugs prompted the search for alternatives. Medicinal plants are a key resource for drug extraction [17]. A number of natural compounds with hepatoprotective properties promising to be safe alternatives to allopathic drugs with less side effects were identified. Three well-characterized examples are the grape skin component resveratrol, green tea catechins (GTCs) and tetrandin, a bis-benzylisoquinoline alkaloid isolated from the medicinal herb *Stephania tetrandra* S Moore. They have been tested for their ability to suppress pro-inflammatory immune responses and/or oxidative stress in ischemic liver [18,19,20].

Trans-resveratrol given before ischemia and before reperfusion at low doses protects against IRI mainly through maintenance of glutathione (GSH) levels and induction of superoxide dismutase (SOD) and catalase (CAT) enzymes [20]. GTCs have antioxidant properties in a dose-dependent manner via maintenance of manganese superoxide dismutase (MnSOD) and suppression of inflammation [19]. The agent tetrandine is anti-inflammatory and blocks calcium-channels. Furthermore, it can reduce oxidative stress by maintaining SOD activity [18].

Yet, in many cases, the actual mechanism of liver protection remains unclear. The agents could act as direct antioxidants, indirectly through nuclear respiratory factor 2 (Nrf2) activation or through unrecognized mechanisms [21]. However, none of the substances was implemented into clinical routine for different reasons. First, the bioavailability of most secondary plant extracts is very low. Second, their activity highly depends on concentration with many having contradictory effects at different concentrations. For instance, resveratrol is a pro-oxidant and may lead to liver damage when given at a high dose [21,22].

A recent study highlighted the hepatoprotective effect of *Antrodia cinnamomea*, a fungus species, in carbon tetrachloride (CCI4) induced hepatic injury in rats. The animals were either orally treated with an *Antrodia cinnamomea* water extract once per day or an emulsified extract for 5 days. The emulsified extract at 50 mg/kg significantly decreased the serum alanine aminotransferase (ALT), aspartate aminotransferase (AST), interleukin-1 (IL-1), IL-6, nitric oxide (NO) and ROS levels. Additionally, cyclooxygenase-2 (COX-2) and caspase-3 protein expression was reduced [23]. This novel and intriguing data strengthens the need for further investigations in this field.

### 1.3. Characterization of Nigella sativa Seeds, Its Oil and Its Active Components

*N. sativa* (Family Ranunculaceae) is a good example for a natural compound with a variety of beneficial properties. The bisexual plant is 20–90 cm tall with finely divided leaves and blue flowers on solitary long peduncles. It forms a fruit capsule which opens up when matured to expose the white trigonal seeds to the air, which then become black in colour [24,25].

*N. sativa* seeds contain numerous esters of unsaturated fatty acids with terpene alcohols. The currently known chemical composition of the seeds is depicted in Table 1.

The oil is one of the key components. An essential and a fixed oil can be extracted from the seeds [24,25,26]. The essential oil ranges from 0.4% to 2.5% [25,27]. It is cold-pressed from the seeds and is a yellow to dark amber liquid. The major active component in the essential oil is thymoquinone (2-isopropyl-5-methyl-1,4-benzoquinone) with up to 50%, followed by p-cymene (40%), pinene (up to 15%) and traces of thymohydroquinone and dithymoquinone.

The fixed oil (36–38%) is produced by hydraulic expression of the seeds [26] and is rich in polyunsaturated fatty acids as well as some minor dihomolinolenic acids, phytosterols and tocopherols [28].

Alpha-hederin, a triterpene saponin, was also identified as an active component of *N. sativa*, but not much has been revealed about its amount in the seeds or its chemical stability. Further constituents of the seeds are unsaturated fatty acids, such as linoleic acid, oleic acid, eicodadienoic and dihomolinoleic acid. About 30% of the seeds consists of saturated fatty acids (stearic acid and palmitic acid) [24,25,29].

### 1.4. Properties of N. sativa

*N. sativa* has a long tradition in the Middle East as a part of an overall holistic approach to health. Commonly known as black seed, *N. sativa* is native to Southern Europe, North Africa and Southwest Asia. It is cultivated in many countries in the world like the Middle Eastern Mediterranean region, South Europe, India, Pakistan, Syria, Turkey and Saudi Arabia [30].

The use is wide spread in various traditional medical systems like Unani-Tibb, Ayurveda, Chinese medicine and Siddha [24]. Among Muslims, it has been defined as an ultimate remedy for all diseases except death due to a Prophetic hadith.

Several in vitro and in–vivo studies were performed to assess the pharmacological effects of *N. sativa* seed and its oil [31,32,33]. Thymoquinone was identified as the active component causing most of the therapeutic effects [25,34] including antioxidant, anti-inflammatory, hepatoprotective, hypoglycemic, anti-hypertensive, analgesic, diuretic, anti-microbial, anti-parasitic, spasmolytic and bronchodilatative properties [34].

### 1.5. Toxicity Studies on N. sativa and Metabolism of TQ

*N. sativa* has a low profile of side effects and high LD_50_ values in experimental studies prove its safety [35,36,37,38].

In 1965, El-Dakhakhani reported the LD_50_ value of TQ as 10 mg/kg when intraperitoneally injected in rats [39]. Later, Mansour et al. indicated, that the LD_50_ value in mice intoxicated with CCI4 is even higher. They reported a LD_50_ dose of 90.3 mg/kg after intraperitoneal (ip) TQ application without specifying the cause of death [40]. In the same study, they did not observe any hepatotoxic effects when applying lower doses (4, 8, 12.5, 25 and 50 mg/kg) TQ. ALT, AST and lactate dehydrogenase (LDH) levels were not significantly elevated upon treatment [39,40,41]. This was confirmed in many other studies. Intraperitoneal TQ doses between 5 to 18 mg/kg were injected to mice and rats without side effects [40,41,42,43,44,45]. However, according to Mansour et al., the only dose that ameliorated hepatotoxicity of CCI4 was 12.5 mg/kg ip and side effects were reported at doses higher than 50 mg/kg ip (see Section 2.2) [40].

Badary et al. reported, that after oral TQ administration in healthy mice the LD_50_ value is even higher (2400 mg/kg). At high doses, signs of toxicity were hypoactivity and difficulty in respiration [38,46]. However, Al-Ali et al. described lower LD_50_ values after oral ingestion in mice (870.9 mg/kg) and in rats (794.3 mg/kg). The animals became more and more drowsy and dyspnoeic before death or they recovered after 24 h. On autopsy, both in the mice and rats receiving lethal doses of TQ, the heart, lungs, liver and kidneys as well as the peritoneum were congested, without any apparent sign of damage or necrosis. Probably, the cause of death was hypotension ultimately leading to shock [35].

Overall, these studies reveal strikingly different results between intraperitoneal and oral TQ administration (>8 to >25-fold difference in dose). The oral application seems to be better tolerated as signs of toxicity occurred after much higher doses than after intraperitoneal application.

According to Wang et al., this may be due to the fact that compounds with low intestinal absorption exhibit less toxicity in oral administration than in the injection route [47]. However, not much is revealed about the metabolism of TQ. Nagi et al. suggest, that TQ may undergo a reduction to dihydrothymoquinone [46]. This theory is strengthened by Mansour et al. They postulate that oral TQ may be transformed into less toxic metabolites in the gastrointestinal tract or into dihydrothymoquinone in the liver. In contrast, when intraperitoneally injected, a complete absorption of TQ into the systemic circulation occurs [40]. Khalife et al. proved, that TQ reacts with GSH, nicotinamide adenine dinucleotide (NADH) and nicotinamide adenine dinucleotide phosphate (NADPH) in physiological conditions. This induces the formation of glutathionyl–dihydrothymoquinone after rapid reaction with GSH and dihydrothymoquinone (DHTQ) after slow reaction time with NADH and NADPH. Interestingly, TQ showed lower scavenging activities than glutathionyl–dihydrothymoquinone and DHTQ. These results suggest, that the cellular antioxidant defense can possibly be modulated through the nonenzymatic metabolic activation of TQ [47].

### 1.6. Objective

Our systematic review aims for providing a comprehensive overview of in vivo experimental and human studies to explore the potential suitability of TQ for reducing hepatic injury in modern surgical treatment concepts.

## 2. Results

**Figure 1:**
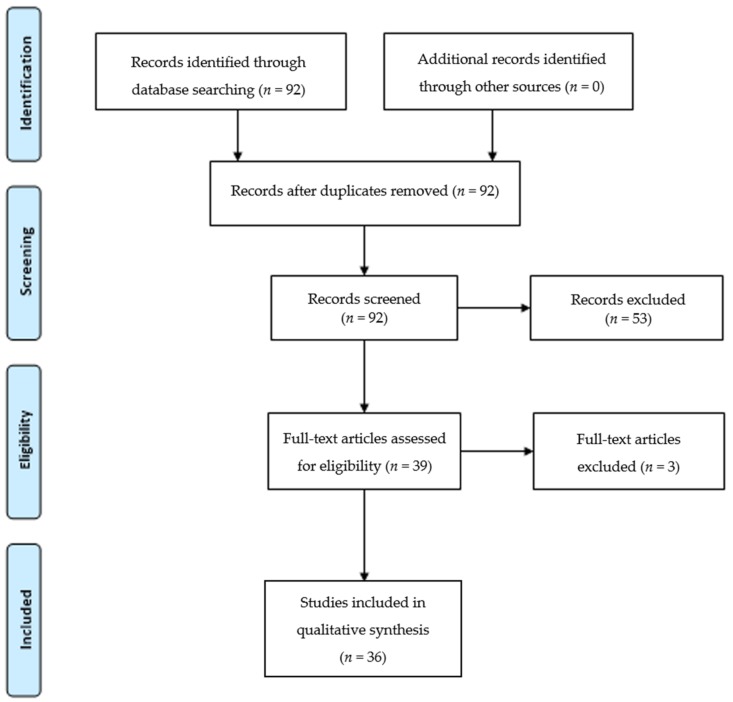
Assessment of eligible articles according to PRISMA 2009 Flow Diagram [48].

The search disclosed a total of 92 articles containing the keywords liver and TQ; 36 studies covering the period from 1999 to 2017 met our selection criteria (Figure 1). The majority of the studies derived from eastern countries (Table 2).

### 2.1. Study Protocols and Applied TQ Doses

We did not find any studies involving humans. The majority of the experiments were performed in rats (*n* = 24; 67%), followed by mice (*n* = 10; 28%) and rabbits (*n* = 2; 6%).

The mainly synthetically manufactured TQ was purchased from chemical companies. In most studies, animals received TQ orally (*n* = 30; 83%). In five studies, TQ was administered intraperitoneally (14%) and in one subcutaneously (3%).

A variety of injury models was used with the majority being hepatotoxic models using drugs such as cyclophosphamide, acetaminophen and methotrexate. Other models employed to investigate the hepatoprotective properties included steatosis, tumor, cholestasis, IRI, healthy animal and irradiation models (Table 3).

A wide range of doses was tested with a minimum of 0.5 mg/kg/day [49,50] and a maximum of 100 mg/kg/day [46,49]. Only Mansour [40] et al. investigated the LD_50_ of TQ in mice intoxicated with CCI4 (see Section 1.5).

### 2.2. Effect of TQ

Our analysis revealed that all authors described a reduction of liver damage in TQ-treated animals, irrespective of the underlying etiology. Liver damage was assessed using histology and liver enzyme release. The beneficial effect was attributed to TQ having strong antioxidant, anti-inflammatory, antifibrotic, anti- and proapoptotic as well as anticarcinogenic effects (Table 4, Table 5, Table 6, Table 7, Table 8, Table 9 and Table 10, see Section 2.2.6).

Side effects were only reported in one study and consisted of oxidative stress leading to hepatic injury when higher doses of TQ (>50 mg/kg ip) were applied [40].

#### 2.2.1. TQ and Oxidative Stress

The antioxidant effect was the most emphasized property of TQ. The majority of the results were achieved in liver toxicity models [40,41,43,44,45,46,50,51,52,53,54,55,56,57,58,59,60,61] and steatosis models [49,62,63], followed by tumor [64] (see Section 2.2.5), cholestasis [65,66], IRI [67,68], irradiation [69] and healthy liver models [70,71].

The liver injury consisted of severe alterations of the liver histology and in significant elevation of liver enzymes such as AST, ALT, ALP and γ-GT. These changes were mediated by an impaired antioxidant system revealed by up- or downregulation of selected oxidative stress markers. As a result of TQ administration, almost all authors reported an improvement of liver histology together with the reduction of liver enzymes. These results suggest that TQ interacts with oxidative stress markers to reinforce the antioxidant system. In detail, upon TQ application the reduction of GSH [45,50,51,53,56,57,58,59,61,67] and NO [53,54,56,67] content, the inhibition of lipid peroxidase (LPO) [43,44,46,51,52,61] as well as the downregulation of malondialdehyde (MDA) [40,43,45,53,55,56,57,59,62,63] were observed.

#### 2.2.2. TQ and Inflammation

In a variety of hepatic injury models, concomitant treatment with TQ led to a reduction of a wide range of different inflammatory markers as described in the following paragraphs.

The majority of the authors attributed an important role to the TQ-induced inactivation of the tumor necrosis factor-α (TNF-α) cell signaling pathway. Within these authors, El-Sheikh et al. [56], Galaly et al. [72], Al-Malki et al. [53], Suddek et al. [61] and Hassanein et al. [58] performed their experiments on toxic hepatic injury models caused by drugs and chemicals, respectively Methotrexate, Gentamicin, Cisplatin, Tamoxifen and Titaniumdioxide nanoparticles. Helal et al. [59] provoked endotoxemia by lipopolysaccharide (LPS) application in the experimental animals. Awad et al. [62] applied a model of nonalcoholic steatohepatitis (NASH) induced by high-fat high-cholesterol diet. Beside the reduction of TNF-α, El-Sheikh et al. also showed a lower level of COX-2 [56]. Al-Malki et al. attributed a further role to the reduction of IL-1β levels [53]. In their steatosis model, Awad et al. reported an increase in IL10 along with TNF-α reduction [62].

Further parameters that seem to be involved in the development of the anti-inflammatory effect of TQ were described by Aras et al. [54], Mohany et al. [44] and Abd-Elbaset et al. [67]. Both, Aras et al. and Mohany et al. applied toxicity models in which the injury was induced by Sodium arsenate (SA) and Imidacloprid, respectively. Abd-Elbaset al. created IRI models through occlusion of the hepatic pedicle for 30 minutes. Aras et al. attributed the favourable effects of TQ to the reduced expression of IL-6, monocyte chemotactic protein-1 (MCP-1) and migration inhibitory factor (MIF) [54]. Mohany et al. explained the improvements in liver histology and blood parameters by increased phagocytic activity, chemokinesis, chemotaxis and immunoglobulin (Ig) levels [44]. Abd-Elbaset et al. showed an association of the anti-inflammatory effects with a decreased myeloperoxidase (MPO) level [67].

#### 2.2.3. TQ and Fibrosis

TQ reduces fibrosis. This was revealed by the normalization of histological changes and liver enzyme levels in toxicity and steatosis models upon application of TQ.

The authors Bai et al., Ghazwani et al. and Yang et al. mainly attributed the positive effect of TQ on fibrotic liver injury to the inhibition of transforming growth factor-β (TGF-ß)-induced hepatic stellate cells (HSC) activation, the downregulation of α-smooth muscle actin (α-SMA) expression and the activation of adenosine monophosphate-activated protein kinase (AMPK) phosphorylation [73,74,75]. The antifibrotic effect of TQ was furthermore associated with the downregulation of collagen-I as described by Bai et al. and Ghazwani et al. [73,74].

Abdelghany et al. attributed the TQ-induced inhibition of the progression of fibrosis in CCI4 toxicity models to the modulation of several fibrosis-related inflammatory mediators such as IL-6, IL-6R, IL-22, IL-22RA1+2, IL-10RA and IL-10RB. In addition, a decrease of TGF-ß1 and TGF-ßRII was analyzed [76].

Furthermore, a significant influence on the breakdown of the extracellular matrix through modulation of metalloproteinases was assigned to TQ. Three different ways were described: upregulation of matrix metallopeptidase 9 (MMP-9) [76], downregulation of tissue inhibitor of metalloproteinase-1 (TIMP-1) mRNA expression [73] and decrease of matrix metallo-proteinase-2 [62] which was observed in NASH-models.

#### 2.2.4. TQ and Apoptosis

Seven authors described the effect of TQ on apoptosis. El-Sheikh et al. [56], Galaly et al. [72], Al-Malki et al. [53], Helal et al. [59], Yang et al. [75] and Ghazwani et al. [74] performed their experiments on chemically induced toxicity models. Awad et al. [62] used a steatosis model.

El-Sheikh et al., Galaly et al. and Helal et al. observed a decrease in caspase 3 expression suggesting lower levels of apoptosis [56,59,72]. The same effect was described by Yang et al., however through enhancement of sirtuin 1 (SIRT1) expression [75]. Pointing in the direction, Galaly et al. and Awad et al. have reported a further antiapoptotic effect via the normalization of proapoptotic Bax expression in cytoplasm and nucleus of hepatocytes as well as the increase of antiapoptotic B-cell lymphoma-2 (Bcl-2) level [62,72].

In contrast, El-Sheikh et al., Al-Malki et al. and Ghazwani et al. found low levels of nuclear factor kappa-B (NF-κB) expression, suggesting a proapoptotic effect [53,56,74].

To summarize, TQ modulates different mechanisms involved in apoptosis and thereby the analyzed effects of TQ apparently vary from anti- to proapoptotic. However, taking into account, that NF-κB is also strongly involved in immune responses and the authors rather concentrated on this topic, the low NF-κB expression should be seen in the context of the anti-inflammatory effect of TQ which dominates the proapoptotic response. 

#### 2.2.5. TQ and Hepatic Malignancy

An important property of TQ is its direct and indirect anticarcinogenic effect in various tumor models. Our focus was on hepatic tumor models.

Ke X. et al. injected Hep3B cells subcutaneously in mice to create HCC models. They observed a normalization of the histology and a decrease of tumor volume when TQ was applied. This effect was explained by inhibition of Bcl-2 expression and repression of the Notch signaling pathway through downregulating NOTCH1 intracellular domain (NICD1). Furthermore, TQ inhibited cell growth by inducing cell cycle arrest through increased expression of p21 protein [77].

In N-nitrosodiethylamine (NDEA) induced HCC rats, Raghunandhakumar S. et al. reported that TQ greatly reduced liver injury markers, decreased tumor markers and prevented hepatic nodule formation through reduced neoplastic transformation of hepatocytes. Application of TQ had an anti-proliferative effect mediated by regulation of the G1/S phase cell cycle transition [78].

Sayed-Ahmed et al. also used NDEA-induced hepatocarcinogenesis models. Pathophysiologically, the initiation of hepatic cancer in these models is induced by decreased mRNA expression of GPx, CAT and GST. TQ application prevented the development of liver cancer by decreasing oxidative stress and preserving the activity and mRNA expression of antioxidant enzymes [64].

In conclusion, these promising properties of TQ can open new perspectives in liver cancer prevention and treatment.

#### 2.2.6. Summary of the Hepatoprotective Effects of TQ in Tables

## 3. Summary

Our systematic review has summarized the effect of TQ on liver injury in experimental studies. This was done to analyze its potential suitability as a liver-protecting drug to improve IRI in transplantation as well as hepatic resection and optimize surgical outcome after hepatotoxic neoadjuvant chemotherapy. So far, no studies regarding the use of TQ in patients have been published despite the strong tradition in several countries.

For the in vivo studies, the key findings can be summarized as TQ having a strong antioxidant, anti-inflammatory, antifibrotic, anti- and proapoptotic and anticarcinogenic effect.

Despite the positive data, there are still some obstacles to be solved. First of all, in the reported studies, a wide range of doses was applied (ranging from 0.5 mg/kg/d [49,50] to 100 mg/kg/d [46,49]). Within this dose range, almost no side effects were reported, suggesting a wide therapeutic window. Yet, explanations of this striking broad dose range might be due to the differences in species and/or the route of administration [35,40,79]. According to toxicity studies (see Section 1.5), oral application of TQ seems to be better tolerated as signs of toxicity occurred at much higher doses than after intraperitoneal application. Overall, the LD_50_ values reported in several studies after both, intraperitoneal injection and oral application, were much higher than the doses of TQ reported to have desirable effects.

Furthermore, as indicated in the reported experiments, TQ modulates various signaling pathways. However, the direct mechanism of action of TQ still remains unclear.

Therefore, for better data reproducibility, but also to improve drug safety and to optimize TQ application for the discussed indications, it is essential to explore the mechanisms and doses inducing benefits and side effects.

## 4. Methods

A systematic search was performed using two databases (Medline and Cochrane Library). The search was last actualized in June 2017. Inclusion criteria were full paper publications in peer reviewed journals reporting original works or systematic reviews.

The search string was (Thymoquinone OR *Nigella sativa* OR *Nigella sat*) AND (hepar OR hepatic OR hep* OR liver) with a filter for language (English) and study settings (humans, animals), without further restrictions.

The title/abstract screening was performed by two of the authors and all publications meeting the inclusion criteria were retrieved as full text. The review was performed in accordance with the PRISMA statement (Figure 1, [48]). The main data were extracted including the publication year, study authors and countries of origin, study settings and design, dose of TQ, LD_50_ dose and route of application.

Furthermore, the outcome of TQ administration in hepatic injury was analyzed by recording the effects of TQ in respect to histological alterations, enzyme release and the postulated mechanisms of action. To classify the main properties, subgroups were defined. The outcomes of the review were not meta-analyzable due to the lack of homogeneity of the data.

## 5. Conclusions

In parallel to the development of novel and effective treatments in modern medicine, the number of drugs with hepatotoxic effects is also increasing. For this reason, substances reducing this toxicity are urgently needed.

Due to the wide therapeutic window with strong antioxidant and anti-inflammatory effects, TQ is a promising drug candidate for IRI. Furthermore, as a hepatoprotective drug with anti-proliferative properties and little reported side effects, TQ could enhance the tolerability and effectivity of neoadjuvant therapy prior to ablative liver surgery.

To date, no attempts have been made to test the potential of TQ at the clinical level. Therefore, a sequence of preclinical and clinical assessments and studies would be adequate.

## Figures and Tables

**Table 1:** Composition of *N. sativa* seeds according to Mollazadeh et al. [25].

Constituent	% Range
Carbohydrates	33–34
Oil	31–35.5
Protein	16–19.9
Fibre	4.5–6.5
Moisture	5–7
Saponins	0.013

**Table 2:** Publication country of first authors and respective number of publications.

Country	*n*
Saudi Arabia	13
China	4
Tunisia	1
Canada	1
Egypt	7
India	2
Malaysia	1
Iran	1
Turkey	4
USA	2

**Table 3:** Hepatic injury models applied in the experiments (*n* = 36).

Model	*n*	%	Corresponding Summary Table
Toxic liver injury	23	64	Table 4
Steatosis	3	6	Table 5
Tumor	3	8	Table 6
Cholestasis	2	6	Table 7
IRI	2	6	Table 8
Healthy liver	2	6	Table 9
Irradiation	1	3	Table 10

**Table 4:** Effects on liver injury caused by intoxication.

	Injury Model		TQ	Effect of TQ						
R	· Model · Duration of Induction · Species	Toxic Effect	· Dose · Route of Administration · Duration of Treatment	Reduction of Hepatic Damage	Anti-Inflammatory	Anti-/Pro-Apoptotic	Antifibrotic	Anti-Carcinogenic	Anti-Oxidative	Others
[56]	· MTX · Single dose (20 mg/kg ip) day 3 · Rat	· Dilatation and congestion of portal vein	· 10 mg/kg/d · po · 10 days	· Normal hepatic architecture	· ↓ TNF-α, COX-2	· ↓ caspase 3 · ↓ NF-kB expression			· ↑ GSH · ↓ MDA, NO, iNOS	
[54]	· SAs · 14 days · Rat	· Elevation of liver enyzmes · ↓ albumin	· 10 mg/kg bw · po · 14 days	· ↓ AST, ALT · ↑ albumin	· ↓ IL-6, MCP-1 and MIF				· ↓ NO	· ↓ TG, creatinine, cholesterol
[72]	· Gentamicin · 21 days (every 2nd day) · Rat	· Hydropic degeneration of hepatocytes, fatty changes, inflammatory cell infiltration, congestion of portal vein · Elevation of liver enyzmes, decrease of proteins	· 20 mg/kg bw · po · 21 days (every 2nd day)	· Normal hepatic architecture · ↓ AST, ALT, LDH, total bilirubin level · improvement of serum total protein, albumin and albumin/ globulin ratio	· ↓ TNF-α	· Caspase 3 and Bax expression reduced to normal · ↑ Bcl-2				
[53]	· Cisplatin · Single injection (12 mg/kg ip) at day 28 · Rat	· Elevation of liver parameters · Vacuolar degeneration and hepatocellular necrosis	· 10 mg/kg/d · po · 28 days pretreatment + 28 days after Cisplatin	· Normal hepatic architecture · ↓ AST, ALT, ALP, γ-GT, total bilirubin, LDH	· ↓ TNF-α and IL-1β	· ↓ NF-kB expression			· ↑ Gpx, GSH, SOD, GST, CAT · ↓ MDA, NO	
[61]	· Tamoxifen · 10 days (45 mg/kg/d ip) · Rat	· Elevation of liver parameters	· 50 mg/kg/d · po · 20 days, starting 10 days before intoxication	· ↓ AST, ALT, LDH, total bilirubin and γ-GT	· ↓ TNF-α				· Normalizing SOD activity · ↓ GSH, LPO	
[44]	· Imidacloprid · 28 days (0.21 mg/kg bw po) · Rat	· Congestion of central vein and blood sinusoids, widely distributed pyknotic nuclei and leukocyte infiltration · Elevation of liver enzymes	· 1 mg/kg · ip · 28 days (every 7th day)	· Improved hepatic architecture · ↓ AST, ALT, ALP	· ↑ phagocyte activity, chemokinesis, chemotaxis, total levels of Ig				· Inhibition of LPO	
[59]	· LPS · Single dose at day 6 · Rat	· Infiltration of inflammatory cells along with necrotic Damages · Elevation of liver enzymes	· 10 mg/kg/d · po · 7 days	· Reduced infiltration of inflammatory cells and necrotic damage · ↓ total bilirubin, ALP, γGT	· ↓ TNF-α	· ↓ caspase 3			· normalization of GSH level · ↓ MDA	
[75]	· Ethanol · 10 days 5% Vol., at day 11 5 g/kg po · Rat	· Hepatocyte enlargement, steatosis, inflammation · Elevation of liver enzymes	· 20 & 40 mg/kg · po · 10 days	· Reduction of histological changes · ↓ ALT, AST		· Enhancement of sirtuin 1 (SIRT1) expression	· Activation of LKB1 and AMPK phosphorylation · Inhibition of TGF-ß induced HSC activation			· ↓ TG
[40]	· CCI4 · Single injection (15 µL/kg ip) · Mouse	· Elevation of liver enyzmes	· 4, 8, 12.5, 25 & 50 mg/kg · ip · pretreatment 1 h before CCI4 injection	· ↓ AST, ALT, LDH					· ↓ MDA · ↑ nonprotein sulfhydryl (-SH) concentration	
[74]	· CCI4 · Single dose on day 6 · Mouse	· Not specified histological changes	· 25 mg/kg/d · po · 7 days	· Significant resistance to liver damage (not specified)		· Inactivation of NF-κB pathway	· Inhibition of upregulation of COL1A1 mRNA expression · Inhibition of HSCs · ↓ α-SMA			
[76]	· CCI4 · 3 µL/g twice weekly for 49 days · Rat	· Cellular infiltration and fibrous septa · Elevation of liver enyzmes	· 35 mg/kg/d · po · 3x/week from day 63 to 119	· Less histological changes · ↓ ALT, AST			· ↓ TGF-ß1, IL-6, IL-22, TGF-ßRII, IL-6R, IL-22RA1+2, IL-10RA, IL-10RB · ↑ MMP9 mRNA			
[43]	· CCI4 · Single injection (20 µL/kg ip) · Mouse	· Elevation of liver enyzmes	· 16 mg/kg/d · po · 1 day (5 days before CCI4 injection)	· ↓ ALT, AST, LDH					· ↑ total SH content · ↓ MDA · Inhibition of LPO	
[46]	· CCI4 · Single injection (20 µL/kg ip) · Mouse	· Elevation of ALT	· 100 mg/kg · po · 1 day	· ↓ ALT					· Inhibition of LPO	
[73]	· Thiocetamide · 3x/week for 35 days · Mouse	· Necrosis, collagen deposition and infiltration of inflammatory cells in liver interstitial · Elevation of liver enyzmes	· 10 & 40 mg/kg · po · 35 days	· Less liver damage, inflammatory cell infiltration, collagen-I and α-SMA positive cells · ↓ ALT, AST			· ↓ protein and mRNA expression of α-SMA, collagen-I, TIMP-1 · AMK phosphorylation			
[52]	· Cyclophos- Phamide · 2 injections (200 mg/kg ip) · Rat	· Elevation of liver parameters	· 10 mg/kg/d · po · 12 days (every 2nd day)	· ↓ ALT, AST, bilirubin, γ-GT					· ↓ LPO	· ↓ urea, creatinine, TG, LDL, cholesterol
[41]	· Cyclophos-phamide · Single dose (250 mg/kg ip) · Mouse	· Severe hemorrhage, necrosis, dilatation of blood vessels · Elevation of liver parameters	· 5 & 10 mg/kg/d · ip · 3 days	· Less hemorrhage and necrosis · ↓ ALT, AST, bilirubin					· ↑ SOD, CAT	
[45]	· Aflatoxin B · Single dose (2 mg/kg ip) · Mouse	· Inflammation, necrosis and hyperlasia of Kupffer cells, infiltration of mononuclear cells, dilatation of sinusoids and disruption of hepatocytes · Elevation of liver enyzmes	· 4, 5, 9 & 18 mg/kg · ip · 3 days pretreatment	· Improvement of histological changes · ↓ AST, ALT, ALP					· ↓ MDA · recovering GSH content	
[60]	· Lead · 35 days · Rat	· Degenerative changes in liver cell architecture · Elevation of liver paramters	· 5 mg/kg/d · po · 35 days	· Prevention of histological changes · ↓ ALT, AST, ALP, γ-GT					· ↑ TAS level	
[58]	· Titanium-dioxide nanoparticles · 42 days · Rat	· Congestion, necrosis and mononuclear infiltration · Elevation of liver enzymes	· 20 mg/kg · po · 42 days	· ↓ ALT	· ↓TNFα				· ↓ oxidative stress, DNA damage · ↑ TAS and GSH levels	
[55]	· Acet-Aminophen · Single dose (500 mg/kg po) · Rat	· Hepatic cell necrosis and toxicity · Elevation of liver enyzmes	· 15 mg/kg/d · po · 1 day	· Less necrosis · ↓ ALT, AST					· ↑ Gpx · ↓ GSSG & SOD activity, serum and tissue MDA levels	
[50]	· Acet-Aminophen · Single injection (500 mg/kg ip) · Mouse	· Elevation of ALT	· 0.5, 1 & 2 mg/kg/d · po · 5 days	· ↓ ALT					· ↓ lipid peroxide, GSH	· ↓ total nitrate/ nitrite, ATP
[51]	· Bisphenol A · 28 days · Rat	· Elevation of liver parameters	· 10 mg/kg · po · 35 days	· ↓ AST, ALT, ALP, γ-GT and level of total bilirubin					· ↓ LPO · normalization of TAS, GSH, Gpx, GST, SOD, CAT	· ↓ total cholesterol · ↓ TG, LDL and vLDL · ↑ HDL
[57]	· Chronic CsA treatment · 28 days · Rat	· Central vein congestion, hepatocyte vaculation, portal inflammation and fibrosis with bile ductular proliferation and focal necrosis · Elevation of liver enyzmes	· 10 mg/kg · po · 28 days	· Mild congestion of central vein and sinuses with minimal fibrotic spots · ↓ AST, ALT					· ↑ GSH, SOD · ↓ MDA	

**Table 5:** Effects on liver injury caused by steatosis.

	Injury Model		TQ	Effect of TQ						
R	· Model · Duration of Induction · Species	Toxic Effect	· Dose · Route of Administration · Duration of Treatment	Reduction of Hepatic Damage	Anti-Inflammatory	Anti-/Pro-Apoptotic	Antifibrotic	Anti-Carcinogenic	Anti-Oxidative	Others
[49]	· Hyper-Cholesterolemia (*cholesterol diet*) · 56 days · Rat	· Elevation of liver parameters	· 20, 50 & 100 mg/kg bw · po · 56 days	· ↓ ALT, γ-GT					· inhibition of OH · ↑ SOD1, CAT, Gpx	· ↓ plasma total cholesterol, LDL cholesterol, creatinine, urea
[62]	· NASH (*cholesterol diet*) · 42 days · Rat	· Focal hepatic necrosis, vacuolation of hepatocytes associated with portal infiltration with inflammatory cells · Elevation of liver enyzmes	· 10 & 20 mg/kg/d · po · 42 days	· TQ 10 mg/kg/day: few inflammatory cells infiltration in between the few fatty changed hepatocytes · TQ 20 mg/kg/day: slight congestion of hepatic sinusoids and minimal vacuolation of sporadic hepatocytes · ↓ AST, ALT	· ↓ TNF-α · ↑ IL-10	· ↓ Bax · ↑ Bcl-2	· ↓ matrix metallo-proteinase-2		· ↓ MDA	· ↓ TG · ↑ HDL
[63]	· Hyper-cholesterolemia · 56 days · Rabbit	· Severe steatosis involving > 66% of hepatocytes, hepatocellular ballooning, mild mixed inflammation · Elevation of liver enyzmes	· 10 & 20 mg/kg/d · po · 56 days	· Reduction of steatosis and inflammation · ↓ AST, ALT					· ↓ protein carbonyl, MDA	· ↓ HDL, LDL, total/HDL-cholesterol

**Table 6:** Effects on liver injury caused by tumor.

	Injury Model		TQ	Effect of TQ						
R	· Model · Duration of Induction · Species	Toxic Effect	· Dose · Route of Administration · Duration of Treatment	Reduction of Hepatic Damage	Anti- Inflammatory	Anti-/Pro-Apoptotic	Antifibrotic	Anti-Carcinogenic	Anti-Oxidative	Others
[77]	· HCC (induced by injection of Hep3B cells sc) · Tumor volume ~150 mm^3^ · Mouse	· Growth of tumor nodules	· 5 & 20 mg/kg/d · sc · 31 days	· ↓ tumor volume				· ↑ expression of p21protein · inhibition of NICD1 and Bcl-2		
[78]	· HCC (induced by NDEA) · Preventive: 16 weeks; Curative: 11 weeks · Rat	· Growth of tumor nodules · Loss of architecture with pleomorphism of nuclei · Elevated liver tumor markers and liver parameters	· 20 mg/kg bw · po · Preventive: 3x/week for 2 weeks prior experiment; Curative: 3x/week for last 5 weeks	· Reduced incidence of tumor nodules · Normal architecture, minimal inflammatory and few neoplastically transformed hepatocytes · ↓ liver tumor markers (AFP & CEA) · ↓ ALT, AST, LDH, ALP, γ-GT, total bilirubin				· ↓ AgNOR · Regulating G1/s phase cell cycle transition		
[64]	· HCC (induced by NDEA) · Single dose NDEA (200 mg/kg ip) · Rat	· Signs of severe hepatic injury (total score: 14) · Elevation of liver parameters	· 4 mg/kg/d · po · 7 days (5 days before, 2 days after NDEA)	· Mild hepatic injury (total score: 6) · ↓ ALT, ALP, total bilirubin					· ↓ thiobarbituric acid reactive substance · ↑ GSH, GPx, GST, CAT	· ↓ total nitrate/ nitrite

**Table 7:** Effects on liver injury caused by cholestasis.

	Injury Model		TQ	Effect of TQ						
R	· Model · Duration of Induction · Species	Toxic Effect	· Dose · Route of Administration · Duration of Treatment	Reduction of Hepatic Damage	Anti-Inflammatory	Anti-/Pro-Apoptotic	Antifibrotic	Anti-Carcinogenic	Anti-Oxidative	Others
[65]	· Bile duct ligation · Rat	· Hepatic necrosis and fibrosis	· 25 & 50 mg/kg · po · 2 weeks (start 3 days prior to ligation)	· Less necrosis and fibrosis					· ↓ HP and MDA · ↑ SOD and Gpx	
[66]	· Bile duct ligation · Rat	· Bile duct proliferation and fibrosis	· 50 mg/kg · po · 2 weeks (start 3 days prior to ligation)	· Attenuation of histological changes					· ↓ tissue hydroxyproline and MDA · ↑ SOD and Gpx	

**Table 8:** Effects on liver injury caused by Ischemia/Reperfusion.

	Injury Model		TQ	Effect of TQ						
R	· Model · Duration of Induction · Species	Toxic Effect	· Dose · Route of Administration · Duration of Treatment	Reduction of Hepatic Damage	Anti-Inflammatory	Anti-/Pro-Apoptotic	Antifibrotic	Anti-Carcinogenic	Anti-Oxidative	Others
[67]	· Occlusion of hepatic pedicule · 30 min · Rat	· Congestion of central vein, dilatation of interstitial spaces, activation of Kupffer cells, hepatocytes with pyknotic nuclei, cytoplasmic degeneration, and inflammatory cell infiltration · Elevation of liver enzymes	· 20 mg/kg/d · po · 10 days	· Moderate congestion of central vein, slight dilation of blood sinusoids · ↓ AST, ALT	· ↓ MPO				· ↓ NO, eNOS, iNOS, NOSTRIN · ↑ GSH	
[68]	· Occlusion of renal artery · 30 min · Rat	· Elevation of ALT	· 10 mg/kg bw · po · 10 days	· ↓ ALT					· ↓ MDA, SSAT mRNA expression · ↑ GST, SOD	· ↓CYP3A1

**Table 9:** Effects on healthy liver.

	Injury Model		TQ	Effect of TQ						
R	· Model · Duration of Induction · Species	Toxic Effect	· Dose · Route of Administration · Duration of Treatment	Reduction of Hepatic Damage	Anti-Inflammatory	Anti-/Pro-Apoptotic	Antifibrotic	Anti-Carcinogenic	Anti-Oxidative	Others
[71]	· Mouse		· 1, 2 & 4 mg/kg/d · po · 5 days						· ↑ GST, quinone reductase	
[70]	· Rabbit		· 10 & 20 mg/kg/d · po · 56 days						· ↑ GST, Gred and Gpx	· ↓ CYP1A, CYP2A4

**Table 10:** Effects on liver injury caused by irradiation.

	Injury Model		TQ	Effect of TQ						
R	· Model · Duration of Induction · Species	Toxic Effect	· Dose · Route of Administration · Duration of Treatment	Reduction of Hepatic Damage	Anti-Inflammatory	Anti-/Pro-Apoptotic	Antifibrotic	Anti-Carcinogenic	Anti-Oxidative	Others
[69]	· 5 Gy gamma · Single dose · Rat	· Not analyzed	· 50 mg/kg/d · ip · 10 days (start 30 min prior irradiation)						· ↓ TOS, OSI, LOOH · ↑ PON	

## Abbreviations

AFP	α-fetoprotein
AgNOR	Argyrophilic nucleolar organizer region
ALP	Alkaline phosphatase
ALT	Alanine aminotransferase
AMPK	Adenosine monophosphate-activated protein kinase
AST	Aspartate aminotransferase
Bcl-2	B-cell lymphoma-2
bw	Body weight
CAT	Catalase
CCA	Cholangiocarcinoma
CCI4	Carbon tetrachloride-induced
CD	Conjugated diene
CEA	Carcinoembryonic antigen
COX-2	Cyclooxygenase-2
CP	Cyclophosphamide
CsA	Cyclosporine A
CYP	Cytochrome
DHTQ	Dihydrothymoquinone
EGF	Epidermal growth factor
Gpx	Glutathione peroxidase
Gred	Glutathione reductase
GSH	Glutathione
GSSG	Glutathione disulfide
GST	Glutathione S-transferase
GTC	Green tea catechin
HCC	Hepatocellular carcinoma
HDL	High density lipoprotein
HP	Hydroxyproline
HSC	Hepatic stellate cells
Ig	Immunoglobulin
IL	Interleukin
IL-1β	Interleukin-1 beta
iNOS	Inducible nitric oxide synthase
ip	intraperitoneal
IR	Ischemia/reperfusion
IRI	Ischemia/reperfusion injury
LD_50_	Mean lethal dose
LDH	Lactate dehydrogenase
LDL	Low density lipoprotein
LOOH	Lipid hydroperoxide
LPO	Lipid peroxidation
LPS	Lipopolysaccharide
MCP-1	Monocyte chemotactic protein-1
MDA	Malondialdehyde
MIF	Migration inhibitory factor
MnSOD	Manganese superoxide dismutase
MPO	Myeloperoxidase
MTX	Methotrexate
NADH	Nicotinamide adenine dinucleotide
NADPH	Nicotinamide adenine dinucleotide phosphate
*N. sativa*	*Nigella sativa*
NASH	Nonalcoholic steatohepatitis induced by high-fat high-cholesterol diet
NDEA	N-Nitrosodiethylamine
NF-κB	Nuclear factor kappa-B
NICD1	NOTCH1 intracellular Domain
NO	Nitric oxide
NOSTRIN	Nitric oxide synthase trafficking
Nrf2	Nuclear respiratory factor 2
OH	Hydroxyl radical
OSI	Oxidative stress index
PCNA	Proliferating cell nuclear antigen
PI3K/Akt	Phosphoinositide 3-kinase
PON	Paraoxonase
R	Reference
ROS	Reactive oxygen species
SAs	Sodium arsenite
sc	subcutaneous
SIRT1	Hepatic sirtuin 1
SOD	Superoxide dismutase
SOS	Sinusoidal obstruction syndrome
SSAT	Spermidine/spermine N-1-acetyl-transferase
TAS	Total antioxidant status
TG	Triglycerides
TGF-β	Transforming growth factor-β
TIMP-1	Tissue inhibitor of metalloproteinase-1
TNF-α	Tumor necrosis factor-α
TOS	Total oxidant status
TQ	Thymoquinone
VEGF	Vascular endothelial growth factor
vLDL	Very low density lipoprotein
α-SMA	α-smooth muscle actin
γ-GT	Gamma-glutamyl transferase
